# Admissions to the Emergency Department Due to Atrial Fibrillation/Atrial Flutter Incidents during the Third Wave of COVID-19 Pandemic

**DOI:** 10.3390/jpm12122003

**Published:** 2022-12-03

**Authors:** Goutam Chourasia, Dorota Zyśko, Joanna Wizowska, Paweł Wróblewski, Katarzyna Madziarska, Kacper Wróbel, Wojciech Timler, Remigiusz Kozłowski, Michał Marczak, Dariusz Timler

**Affiliations:** 1 Department of Emergency Medicine, Wroclaw Medical University, 50-556 Wroclaw, Poland; 2 Department of Emergency Medical Service, Wroclaw Medical University, 50-556 Wroclaw, Poland; 3 Department of Nephrology and Transplantation Medicine, Wroclaw Medical University, 50-556 Wroclaw, Poland; 4 Department of Management and Logistics in Healthcare, Medical University of Lodz, 90-419 Lodz, Poland; 5 Department of Emergency Medicine and Disaster Medicine, Medical University of Lodz, 90-419 Lodz, Poland

**Keywords:** atrial fibrillation, atrial flutter, COVID-19, emergency department, SARS-CoV-2

## Abstract

(1) Background: Severe acute respiratory syndrome coronavirus 2 (SARS-CoV-2) infection increases the risk of atrial fibrillation/flutter (AF/AFL) incident. The study aimed to present the characteristics of admissions to the emergency department (ED) due to AF/AFL incidents during the third COVID-19 pandemic wave. (2) Methods: A retrospective analysis of the medical records of the ED patients: 8399 during 3 months of the second and 11,144 during the 3 months of the third pandemic wave. (3) Results: SARS-CoV-2 positive patients there were 295 (3.5%) during the second wave and 692 (6.2%) during the third wave (*p* < 0.001). Among patients with SARS-CoV-2 infection, there were 44 (14.9%) patients with known AF/AFL during the second wave and 75 (10.8%) during the third wave, respectively (0.07). There were 116 visits with a diagnosis of AF/AFL incident during the third wave (study group) and 76 visits during the second wave (control group). The SARS-CoV-2 test was positive in 11 (9.5%) visits in the study group and in 1 (1.3%) visit in the control group *p* = 0.047. During the third wave, the patients with AF/AFL incidents with positive tests were older and more often had new-onset AF/AFL than those with negative tests: 76.3 (13.2) years vs. 71.8 (12.6) years; and 4 (36.4%) patients vs. 7 (7.6%) patients, respectively. (5) Conclusions: During the third pandemic wave, the number of patients with SARS-CoV-2 infection increased in comparison to the second wave. Additionally, among patients with AF/AFL incidents, the percentage of SARS-CoV-2-positive patients increased. During the third wave, the patients with positive tests and AF/AFL incident were older and more often had new-onset AF/AFL than those with AF/AFL incident and negative test which indicate the arrhythmogenic effect at the onset of the disease, especially in the older population.

## 1. Introduction

Atrial fibrillation and atrial flutter (AF/AFL) occurs in 3–10% of the patients admitted to the emergency department (ED) [1]. The AF/AFL incident was the direct cause of admission to ED in 1% of the patients [2]. During the second wave of the COVID-19 pandemic, the number of all ED visits and visits due to AF/AFL incidents decreased in comparison to the non-pandemic era [3,4,5,6].

AF/AFL is common among hospitalized patients with COVID-19 disease and predicts a worse prognosis [7]. In the meta-analysis of Romiti et al., the prevalence of new-onset AF defined as presenting with AF during COVID-19-related hospitalization, with the exclusion of those with a previous history of AF, was 7.4%. [8]. It is presumed that patients with COVID-19 and newly diagnosed AF may have a preexisting substrate for AF and SARS-COV-2 infection is a trigger for AF initiation [9].

A detailed analysis of the pathogenesis of AF in patients with COVID-19 was presented by Stone et al. The electrical perturbances at the tissue and cellular level could be related to the dysfunction of microvascular support by pericytes or endothelial cells, fibrosis, increased tissue edema, and interstitial hydrostatic pressure [10].

The infection may be a provoking factor for AF/AFL occurrence. It may also result in an increased heart rate of chronic AF [11]. Both AF/AFL incidents and an increased rate of chronic AF may prompt a patient to attend the ED even if the other symptoms of SARS-CoV-2 infection are not present. On the one hand, among patients admitted to the ED with the main problem presumed to be the AF/AFL, incidentally, some patients may have SARS-CoV-2 infection. On the other hand, in patients with COVID-19 disease, the other symptoms are usually more serious, and the AF/AFL incident is not the main diagnosis. During the second wave of the COVID-19 pandemic in Poland, the patients with AF/AFL as the main diagnosis had concurrent SARS-CoV-2 infection in less than 1% of the cases [4]. However, the second wave was not as bad and therefore, the effect of the SARS-CoV-2 infection on the occurrence of AF/AFL as the main diagnosis could not be revealed. The third wave of the COVID-19 pandemic occurred in Poland in early March 2021 with a peak in April/May. During this period, the number of diagnosed SARS-CoV-2 infections was up to 30,000 per day [12]. The AF/AFL incident occurrence at the ED may also be assessed in the context of recent vaccinations introduced since December 2020.

The aim of the study was to present the clinical characteristics of the patients admitted to the ED due to AF/AFL incidents during the third wave of the COVID-19 pandemic. Furthermore, the vaccination status of the patients was presented.

## 2. Materials and Methods

### 2.1. Study Design

The study was designed as a retrospective analysis of the medical records of the patients admitted to the Emergency Department of the University Hospital between 1 March and 31 May 2021 (study period) and between 1 October and 31 December 2020 (reference period).

The electronic medical recordings were searched to find admissions related to AF/AFL incidents during study and reference periods, admissions of patients with positive SARS-CoV-2 swab tests during study period, and admissions related to the occurrence or risk of post-vaccination complications during the study period. The first search was performed to find patients with AF/AFL incidents. The visits with ICD-10 code I48, code R02, or the words “palpitations” and “atrial fibrillation” in the medical records were retrieved. The retrieved cases were manually checked to choose patients in whom the AF/AFL incident was considered to be the main cause of the admission. The group was assigned AF/AFL (+).

The second search was performed to find SARS-CoV-2-positive patients during study period using a code U07.1. These patients were assigned SARS-CoV-2 (+). 

The third search was performed to find patients with dyspnea as a complaint at admission.

For the patients who were recognized to be admitted because of AF/AFL incidence, the following data were retrieved from the electronic recordings: age, sex, how the patients reached the ED, the duration of AF/AFL incident, the chosen strategy of rate or rhythm control in the ED, the used antiarrhythmics, the performed electrotherapy, and the mode of discharge (admission to the other ward or home discharge, the occurrence of positive SARS-CoV-2 test, the previous full vaccination (the admission later than two weeks after the appropriate vaccination).

The complaints reported at admission to the ED by patients with AF/AFL incidents as well SARS-CoV-2 positive patients during the study period were noted as follows: palpitations, altered mental status, dyspnea, fever, cough, chest pain, gastrointestinal symptoms, vertigo, muscle pain, and headache. 

The descriptions of the ECGs of patients with positive SARS-CoV-2 swab tests were checked to find the patients with chronic AF, paroxysmal AF in the medical history but sinus rhythm at admission, and paroxysmal AF at admission.

The age, sex, and vaccination status of SARS-CoV-2-positive patients were also noted.

The last search of the medical electronic records was performed to find patients who complained of or were afraid of possible postvaccination complications using words “postvaccination” and “complications” and chosen subjects had their medical records manually checked to find those with AF/AFL incidents.

The Emergency Department of Wroclaw University Hospital covers about 200,000 inhabitants and this number was used in calculations regarding the occurrence of AF/AFL incidents.

### 2.2. Statistical Analysis

The study was approved by Bioethical Commission of Wroclaw Medical University (KB 850/21). 

The continuous variables with normal distribution were presented as mean and standard deviations and compared with Student‘s T-test. The continuous variables with non-normal distribution were presented as median and interquartile ranges (IQR) and compared with Mann–Whitney U test. 

The discrete variables were presented as numbers and percentages and compared with chi-squared test. 

*p* less than 0.05 was considered significant.

## 3. Results

During the study period, there were 11,144 admissions to the ED and there were 116 admissions with AF/AFL incidents as the main cause of the admission (study group). During the reference period, there were 8399 admissions to the ED. Among them, there were 76 visits with AF/AFL incidents as the main cause of the admission (control group).

During the reference period, there were 295 (3.5%) SARS-COV-2-positive patients and during the study period, there were 692 SARS-CoV-2-positive patients (6.2%) (*p* < 0.001).

Among the Sars-CoV-2-positive patients, 44 (14.9%) patients had known AF/AFL during the reference period and 75 (10.8%) patients during the study period *p* = 0.07)

The total number of patients presenting during the reference period and during the study period with known AF/AFL was, respectively, 578 (6.9%) and 623 (5.5%) patients (*p* < 0.001).

The new onset AF/AFL among SARS-CoV-2 positive patients was found in no patients during the reference period and in 4 patients in the study period.

Dyspnea as a main complaint was noted in 542 (6.5%) of the patients during the reference period and 900 (8.1%) during the study period *p* < 0.001

There were 192 visits due to AF/AFL incidents in 152 patients in total during the study period and the reference period. The patients were 72.4 ± 13.3 of age and there were 70 men aged 66.2 ± 13.2 and 82 women aged 77.7 ± 11.0 *p* < 0.001.

The positive SARS-CoV-2 swab test was found in 11 (9.5%) visits of the study group and 1 (1.3%) visit of the control group *p* = 0.047.

In Table 1, the comparison of patients admitted due to AF/AFL incidents during the study and reference period was presented.

The patients with AF/AFL incident as the main diagnosis in the ED during the study period are less likely to be brought by emergency medical services and do not differ in terms of the other studied parameters.

In Table 2, the main complaints of the patients admitted during the study period with the diagnosis at the discharge of COVID-19 disease without recognized AF/AFL incident as a chief complaint, with diagnosis at the discharge of COVID-19 disease with recognized AF/AFL incident as a chief complaint, and diagnosis of AF/AFL incident with negative SARS-CoV-2 were compared.

### The Vaccination Status of the Patients with AF/AFL Incident during Study Period

Full vaccination before admission to the AF/AFL event had 27 (23.3%) patients with AF/AFL during the study period and 3.6% of patients with positive SARS-CoV-2 swab test. All the patients with AF/AFL incidents and positive swab tests were not vaccinated before the ED admission or received only the first dosage of the vaccine (in 3 cases).

Among patients with positive SARS-CoV-2 test with the chief complaint at admission other than related to AF/AFL incident 14 (2.1%) had atrial fibrillation incident, 41 (6.0) had chronic atrial fibrillation and 14 (2.1%) had sinus rhythm and AF/AFL in their medical history. Therefore, among a total of 692 (681 plus 11) patients with positive SARS-CoV-2 test at admission to the ED, there were 80 patients (11.5%) with current or previous AF/AFL. 

None of the 137 patients admitted because of the complaints about the risk or the presence of postvaccination complications had AF/AFL incidents.

## 4. Discussion

The first finding of the study is that the number of admissions to the ED due to SARS-COV-2 infection increased during the study period in comparison to the reference period. In addition, the number of AF/AFL incidents during the third wave of the COVID pandemic increased in comparison to the second wave. However, among patients with positive SARS-CoV-2 test, the percentage of patients with known AF/AFL decreased. This could be related to the increased mortality of patients with numerous comorbidities during earlier pandemic waves.

The earlier reports indicated that the number of patients admitted to the ED during the early pandemic period was significantly lower than during the pre-pandemic period [4]. 

In the study by Bilaszewski et al., the number of admissions due to AF incidents was 232 during six months (mean of 39 events per month). During six months of the second wave of the COVID-19 pandemic, the number of ED visits due to AF events was 157 (mean of 26 events per month). Decreases in ED visits during the early phase of the pandemic were a common phenomenon. Furthermore, the number of ED visits due to AF episodes also decreased during the early phase of the COVID-19 pandemic [4]. This decrease was considered to be caused by the reluctance of the patient to attend the ED because of the fear of infection. Additionally, the decrease in AF events was considered. The return to the previous level of admissions due to AF episodes during the third wave of the COVID-19 pandemic indicates the former cause is more probable. Nonetheless, the return of the AF/AFL incident visits to the pre-pandemic level does not mean a lack of fear to attend the ED. The median time of the AF/AFL incident during the third wave was 9 h which is still longer than 5 h in the pre-pandemic period in the same hospital as reported by Bilaszewski et al. [4]. This finding indicates that the patients are still reluctant to be treated in the ED. 

The second finding is that the number of patients with AF/AFL incident as the chief diagnosis at the time of admission and concomitant COVID-19 infection increased during the third wave of the COVID-19 pandemic. These patients did not present with typical infection symptoms such as fever or cough and therefore, the recognition of the COVID-19 infection was unexpected. However, the patients with confirmed SARS-CoV-2 infection very rarely had palpitations as a chief complaint [7]. The obtained results are in line with the reports that the most common symptoms at the admission of patients with COVID-19 disease are fever, cough, dyspnea, and gastrointestinal symptoms [7,12]. The new onset AF/AFL incident was more common in SARS-CoV-2 positive patients than negative patients during the third pandemic wave. The observation indicates the arrhythmogenic effect of the SARS-CoV-2 infection.

COVID-19 disease is a serious infection with an increased risk of death due to a hypercoagulable state, hyperinflammatory cytokine storm, and multiorgan failure. AF/AFL is frequent in patients with COVID-19 disease. The history of AF/AFL was positive in 23.8% of the deceased and 8.8% of survivors. AF/AFL was recognized on the ECGs from 6.2% of patients with COVID-19 disease admitted to the ED [13,14]. In a substantial percentage of patients, it occurs during the disease and may worsen the outcome [11,15,16,17]. The occurrence of AF/AFL during COVID-19 disease may be related to the electrolyte disturbances which occur frequently in these patients, hemodynamic compromise, and treatment with catecholamines [18]. However, in these cases, arrhythmia is not a dominant symptom. Therefore, in a patient with complaints of sudden onset palpitations and no other symptoms such as fever, cough, and gastrointestinal problems, COVID-19 disease is less likely but is not impossible.

During the third wave of the COVID-19 epidemic, the vaccination program started. It was found that among patients with positive swab tests that only 5% of patients were vaccinated. Contrary, in the group of patients with AF/AFL incident and negative swab test there were 27% of vaccinated patients which confirms the beneficial effects of vaccination against SARS-CoV-2 infection. The presented data may be used for breaking COVID-19 vaccine hesitancy which is observed in Poland as in other countries [19]. Furthermore, during the study period, there were no patients admitted with AF/AFL incidents who claimed postvaccination complications. This finding is in line with the data reported by Klugar et al. who found that the most common side effects of vaccination against COVID-19 are local side effects, related to the injection site, headache/fatigue, muscle pain, malaise, chills, and joint pain [20].

### Limitations

The main limitation of the study is its retrospective design and single-center nature. Furthermore, the sample size is low. Moreover, during the pandemic, the potential bias due to underdiagnosis of cases could occur especially due to the tendency to shorten the contact with the patients and limit contact with the patient’s families. Finally, the data are medical records about AFL/AFL, which did not allow us to distinguish with certainty whether the AF/AFL in known patients was paroxysmal, persistent, or chronic. Furthermore, the number of new-onset AF/AFL incidents was low. On the one hand, its percentage is in line with expectations, but on the other hand, the differences should be interpreted with caution. However, the analyzed data came from the records performed at the time of the patients’ admissions. Especially, the data recorded by trialists were noted before the laboratory findings were available. 

The other limitation is that not all patients admitted to ED had PCR tests against SARS-CoV-2 performed.

## 5. Conclusions

During the third pandemic wave, the number of patients with SARS-CoV-2 infection increased in comparison to the second wave. Additionally, among patients with AF/AFL incidents, the percentage of SARS-CoV-2-positive patients increased. During the third wave, the patients with positive tests and AF/AFL incidents were older and more often had new-onset AF/AFL than those with AF/AFL incidents and negative tests which indicates the arrhythmogenic effect at the onset of the disease, especially in the older population.

## Figures and Tables

**Table 1:** The comparison of the patients admitted to the ED due to AF/AFL incidents admitted during the study and the reference period.

	Study Period AF/AFL (+) N = 116	Reference Period AF/AFL (+) N = 76	*p*
Age.; mean (SD)	72.3 (12.1)	71.2 (14.9)	0.581
Male sex.; n (%)	52 (46.6)	45 (59.2)	0.051
AF/AFL duration.; h.; median (IQR)/# of known	9 (5–48)/82	10 (4–60)/48	0.341
Unknown or unrecorded duration of AF.; n (%)	34 (29.;3)	28 (36.8)	0.275
Sinus rhythm restoration before the ED admission n (%)	11 (9.5)	4 (5.3)	0.287
Sinus rhythm at the ED discharge	57 (51.1)	37 (48.7)	0.951
Hospital admission n (%)	28 (24.1)	12 (15.8)	0.164
New onset n (%)	11 (9.5)	7 (9.2)	0.950
Rate control strategy at the ED n (%)	35 (30.2)	30 (39.5)	0.183
Phenazoline.; n (%)	24 (20.7)	24 (32.0)	0.089
Amiodarone.; n (%)	13 (11.2)	7 (9.3)	0.658
Beta-blocker.; n (%)	31 (26.7)	13 (17.3)	0.121
Rytmonorm n (%)	5 (4.3)	3 (4.0)	0.902
Electrocardioversion.; n (%)	7 (6.0)	8 (10.7)	0.257
SARS-CoV-2-positive n (%)	11 (9.5)	1 (1.3)	0.047
Brought to the ED by EMS n (%)	57 (67.8)	54 (71.1)	0.002
Duration of the stay in the ED (min) median (IQR)	413.0 (283.5–564.0)	435.5 (297.5–634.0)	0.436

**Table 2:** Main complaints at the time of admission to the ED.

	SARS-CoV-2 (+) AFL/AF (-) (1) N = 681	SARS CoV-2 (+) AFL/AF (+) (2) N = 11	SARS CoV-2 (-) AFL/AF (+) (3) N = 105	*p* (1) vs. (2)	*p* (1) vs. (3)	*p* (2) vs. (3)
Age.; years.; mean (SD)	60.6 (18.1)	76.3 (13.2)	71.8 (12.6)	0.001	0.001	0.001
Male gender.; n (%)	353 (51.8)	2 (18.2)	50 (47.6)	0.053	0.389	0.121
Palpitations.; n (%)	5 (0.7)	5 (45.5)	93 (88.6)	<0.001	<0.001	<0.001
Fever.; n (%)	247 (36.3)	0 (0)	0 (0)	0.030	0.001	0.968
Altered mental status.; n (%)	111 (16.3)	0 (0)	6 (5.7)	0.295	0.005	0.921
Dyspnea.; n (%)	389 (56.3)	6 (54.4)	18 (17.1)	0.868	<0.001	0.012
Cough.; n (%)	182 (26.7)	0 (0)	0 (0)	0.099	<0.001	0.999
Gastrointestinal problems.; n (%)	97 (14.3)	0 (0)	4 (3.8)	0.362	0.005	0.834
Chest pain.; n (%)	40 (5.9)	3 (27.3)	13 (12.4)	0.034	0.013	0.366
New onset AF.; n (%)	4 (0.6)	4 (36.4)	7 (6.7)	0.001	0.002	0.008
Full vaccination at least 14 days before positive SARS-CoV-2 test n (%)/N N-the number of patients with known vaccination history	18 (3.7)/490	0 (0)/11	27 (25.7)/105	0.517	<0.001	0.123

## Data Availability

The data presented in this study are available on request from the corresponding author. The data are not publicly available due to privacy issues.
